# Exercise modulates behavioral and neural mechanisms of working memory in excessive short video users

**DOI:** 10.3389/fpsyg.2026.1875248

**Published:** 2026-07-06

**Authors:** Tian Feng, Youxin Wei, Yawei Li

**Affiliations:** 1 Department of Physical Education, Henan Sport University, Zhengzhou, Zhengzhou, Henan, China; 2 School of Physical Education, Henan University, Kaifeng, Henan, China; 3 School of Kinesiology and Physical Education, Zhengzhou University, Zhengzhou, Henan, China; 4 Department of Sports, Henan Sport University, Zhengzhou, Henan, China; 5 School of Physical Education and Sport Science, Fujian Normal University, Fuzhou, China

**Keywords:** brain activation, cognitive efficiency, exercise, short video, working memory

## Abstract

**Objective:**

This study examined the associations between different exercise habits, working memory performance, and prefrontal cortical activation patterns in male college students with excessive short video use.

**Methods:**

Eighty-two male college students were recruited. Behavioral tests and functional near-infrared spectroscopy (fNIRS) were used to assess working memory performance and prefrontal oxygenated hemoglobin (OxyHb). Participants were classified by daily exercise habits (high exercise habits [HE], low exercise habits [LE], no exercise habits [NE]) and short video usage (low video [LV], moderate video [MV], high video [HV]).

**Results:**

(1) In working memory tasks, the LV group exhibited significantly shorter reaction times and higher accuracy ratio than the MV and HV groups (all *p* < 0.01). The MV group also outperformed the HV group (all *p* < 0.05). (2) The HE group demonstrated higher accuracy and accuracy ratio than both the LE and NE groups (*p* = 0.035), and the LE group performed better than the NE group (*p* < 0.05). (3) A significant interaction between exercise habits and video usage duration was observed in channels 3 (left ventrolateral prefrontal cortex [VLPFC]), 6 (frontopolar cortex [FPC]), and 11 (orbitofrontal cortex [OFC]) during working memory tasks (all *p* < 0.05).

**Conclusion:**

Excessive short video use was associated with poorer working memory performance, whereas regular exercise habits were associated with better behavioral performance. Moreover, exercise habits and short video use interacted to modulate prefrontal OxyHb in regions critical for cognitive control and decision-making. These findings suggest that promoting regular physical exercise may help counteract the negative cognitive effects of excessive short video consumption.

## 1 Introduction

Excessive use of short videos has become a pressing social issue (Chern and Huang, 2018; Milková and Ambroová, 2018). As of March 2025, the number of short video users reached 1.04 billion, and the average person used it for 156 min a day (China Internet Network Information Center, 2025). Short video platforms, through their precise algorithmic recommendation mechanisms and vast amounts of fragmented content, induce users to engage in high-frequency, immersive overuse and dependency (Chou and Hsiao, 2000). Excessive short video use refers to an individual’s inability to effectively control viewing behavior, which can negatively impact cognitive functions (Ye et al., 2023). According to information overload theory, during excessive short video consumption, the brain must allocate more cognitive resources to continuously process excessive visual and auditory information, leading to overload. This results in reduced accuracy in encoding, storing, and retrieving information, thereby impairing cognitive functions (Lee et al., 2016). Notably, the Oxford Word of the Year “Brain Rot” captures the phenomenon of potential degradation in human intelligence and mental health in the information age, emphasizing the detrimental effects of excessive exposure to low-quality, fragmented information on cognitive and psychological wellbeing (Oxford University Press, 2024). Therefore, exploring solutions to mitigate the cognitive impairments caused by excessive short video use has become imperative.

As a core element of cognitive ability, working memory critically determines an individual’s mental activities and behavioral performance when dealing with complex tasks (Wu et al., 2018). Research on internet use has revealed a significant relationship between excessive usage and working memory decline, as measured by reaction time and accuracy (Kandjani et al., 2020; Zhou et al., 2016). However, systematic research on short video use remains insufficient; in particular, studies focusing on excessive short video use to explore its relationship with and mechanisms affecting working memory are relatively scarce. Multiple studies have confirmed that physical exercise beneficially regulates brain cognitive functions and counteracts working memory decline (Erickson et al., 2009; Cai and Zhang, 2019). A study examine the concurrent performance of working memory and cortical activity during acute aerobic exercise in young adults and the fNIRS results showed that the oxygenated hemoglobin (oxy-Hb) concentrations in the bilateral frontopolar area, dorsolateral prefrontal cortex, and right premotor and supplementary cortex were decreased while cycling (Zheng et al., 2022).

While the benefits of physical exercise for working memory are well established, less is known about whether different exercise habits exert differential effects on working memory ability, specifically in the context of working memory decline induced by excessive short video use. In other words, does the amount—rather than merely the type—of regular physical activity determine the degree of protection against cognitive impairment? Existing evidence suggests that individuals with higher levels of daily exercise habits tend to show better cognitive performance and greater neural efficiency than those with low or no exercise habits (Chen et al., 2016). Additionally, research on internet addiction has proposed a close relationship between physical exercise status and addictive tendencies (Di Nicola et al., 2017), indicating that individuals who engage in insufficient or inactive physical exercise are more prone to developing internet addiction(Khan et al., 2017). Based on these findings, long-term exercise habits may hold significant value in regulating and improving working memory ability in excessive short video users.

As a non-invasive and convenient neuroimaging technique, functional near-infrared spectroscopy (fNIRS) has become an important tool for monitoring cerebral oxygenation and hemodynamic changes during working memory processes (Herold et al., 2020). The prefrontal cortex (PFC) is a key brain region associated with working memory, and physical exercise enhances blood flow directed toward the PFC (Purohit and Pradhan, 2017). Functional imaging studies have revealed the activation mechanisms of the PFC during working memory-related tasks (Mckendrick et al., 2020; Zhang et al., 2021), as well as how physical exercise promotes catecholamine secretion and increases blood flow, thereby enhancing PFC activity and strengthening functional network connectivity among related brain regions. Therefore, the present study used fNIRS to monitor hemodynamic changes in the PFC, explore the regulatory effects of physical exercise on brain processing patterns of working memory in excessive short video users, and analyze alterations in hemodynamic changes during task performance.

In summary, this study investigated the brain processing mechanisms through which different levels of physical exercise habits regulate working memory in excessive short video users from both behavioral and cerebral hemodynamic perspectives, thereby explaining differences in behavioral performance and oxygenated hemoglobin (OxyHb) levels associated with varying exercise habits. The aim is to advance theories on how regular physical exercise delays working memory decline and to deepen the scientific understanding of the psychological benefits of physical exercise. Based on the above reasoning, we hypothesize that higher levels of exercise habits are associated with better behavioral performance in working memory processing among excessive short video users and with more efficient brain activation patterns during working memory tasks.

## 2 Methods

### 2.1 Subjects

This study recruited male college students as participants. Inclusion criteria were: (1) no history of cardiovascular disease or sports injuries; (2) no concurrent participation in other experiments affecting cognitive or neurological functions. Exclusion criteria were: (1) history of neurological or psychiatric disorders; (2) uncorrected visual or auditory impairment. To identify individuals with problematic short video use, we administered the Problematic Short Video Use Scale (Mao et al., 2022). This 13-item scale uses a 5-point Likert scale, with total scores ≥39 indicating problematic use. In this sample, the scale demonstrated good internal consistency (Cronbach’s *α* = 0.839).

A total of 500 electronic questionnaires were distributed, and 436 valid responses were returned. After applying the screening criteria and the scale cutoff score (≥39), 262 college students who met the criteria for excessive short video use were identified.

To eliminate self-report bias, we collected official mobile app records from all 262 eligible participants: (1) short video usage: 1-month continuous daily screen time data (video playback only) from built-in mobile system usage statistics; (2) exercise habits: Three-month continuous exercise frequency and duration records from built-in health apps or fitness tracking apps (e.g., Keep, Apple Health), excluding mandatory physical education classes. A total of 180 participants were excluded at this stage due to: (1) Not having enabled app usage tracking function (*n* = 72). (2) Incomplete short video recording data for less than 1 month (*n* = 56). (3) Incomplete exercise recording data for less than 3 months (*n* = 52). This left 82 participants with complete and valid objective data for both short-video use and exercise habits. Then, participants were classified using a two-step hierarchical grouping method based on their objectively verified data:

First step: short video usage grouping. All 82 excessive-use participants were ranked by their average daily short video screen time. Following Kelley’s extreme grouping method (Kelley, 1939), participants were divided into three groups: (1) Low video (LV) group: bottom 27% (*n* = 28, 2.16 ± 0.54 h/d). (2) Moderate video (MV) group: middle 46% (*n* = 27, 4.24 ± 0.67 h/d). (3) High video (HV) group: top 27% (*n* = 27, 6.17 ± 0.87 h/d).

Second step: Exercise habits grouping within each video group. Within each of the three video usage groups, participants were further classified into three exercise habit groups based on their objectively verified exercise frequency and duration over the past 3 months:(1) High exercise (HE) group: ≥3 sessions/week, ≥1 h/session. (2) Low exercise (LE) group: 1–2 sessions/week, ≥1 h/session. (3) No exercise (NE) group: ≤1 session/week.

This study employed a 3 (exercise: HE, LE, NE) × 3 (video usage: LV, MV, HV) between-subjects factorial design. The hierarchical grouping yielded the following subgroup sizes (Table 1):

**Table 1:** Information of the participants.

Group		Number/people	Reaction time	Accuracy	Accuracy ratio
NE	LV	9	771.51 ± 138.82	0.78 ± 0.14	1.08 ± 0.27
	MV	8	824.6 ± 73.31	0.81 ± 0.11	1.01 ± 0.15
	HV	9	847.11 ± 87.42	0.85 ± 0.09	1.02 ± 0.15
	Total	26	827.13 ± 86.06	0.83 ± 0.11	1.02 ± 0.16
LE	LV	9	721.58 ± 94.48	0.89 ± 0.08	1.28 ± 0.21
	MV	10	801.53 ± 100.35	0.87 ± 0.06	1.1 ± 0.16
	HV	9	877.88 ± 70.74	0.87 ± 0.07	1.02 ± 0.16
	Total	28	789.34 ± 107.3	0.87 ± 0.07	1.14 ± 0.2
HE	LV	10	696.41 ± 90.94	0.93 ± 0.02	1.44 ± 0.3
	MV	9	737.32 ± 61.49	0.92 ± 0.04	1.25 ± 0.14
	HV	9	851.96 ± 80.61	0.9 ± 0.04	1.07 ± 0.13
	Total	28	755.75 ± 92.69	0.92 ± 0.04	1.25 ± 0.22
LV		28	720.26 ± 97.43	0.88 ± 0.08	1.3 ± 0.27
MV		27	786.17 ± 85.82	0.87 ± 0.09	1.13 ± 0.18
HV		27	856.61 ± 78.79	0.87 ± 0.07	1.03 ± 0.14
total		82	789.85 ± 99.18	0.87 ± 0.08	1.14 ± 0.22

HE, high exercise habits; LE, low exercise habits; NE, no exercise habits; LV, low short video usage; MV, moderate short video usage; HV, high short video usage. Values are means ± SD.

**Figure 1:**
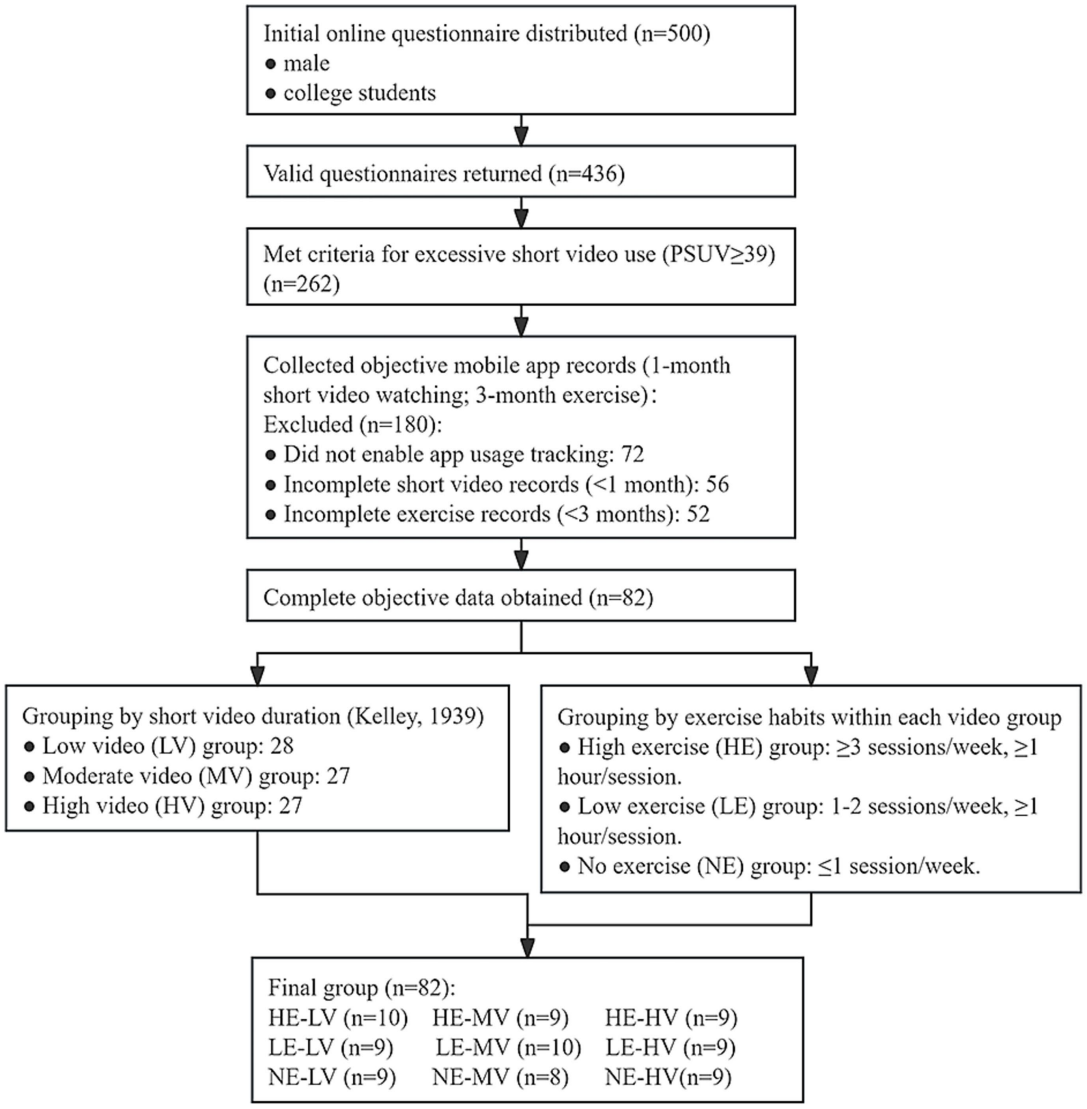
Participant flow diagram.

The final sample consisted of 82 participants. *Post-hoc* power analysis using G*Power 3.1 (effect size *f* = 0.4, *α* = 0.05, *N* = 82) yielded a power of 0.87, exceeding the recommended threshold of 0.80. No significant age differences were observed across the nine groups, *F*(8, 73) = 0.426, *p* > 0.05. Independent-samples *t*-tests revealed no significant differences between the included (*n* = 82) and excluded (*n* = 180) participants in age, *t*(260) = 0.52, *p* = 0.603, excessive short video use scale scores, *t*(260) = 0.71, *p* = 0.478, or daily short video duration, *t*(260) = 0.41, *p* = 0.682, suggesting that selection bias is unlikely to have substantially affected the results. All participants provided written informed consent before the experiment. This study was approved by the university’s human research ethics committee (Figure 1).

### 2.2 Experimental task

**Figure 2:**
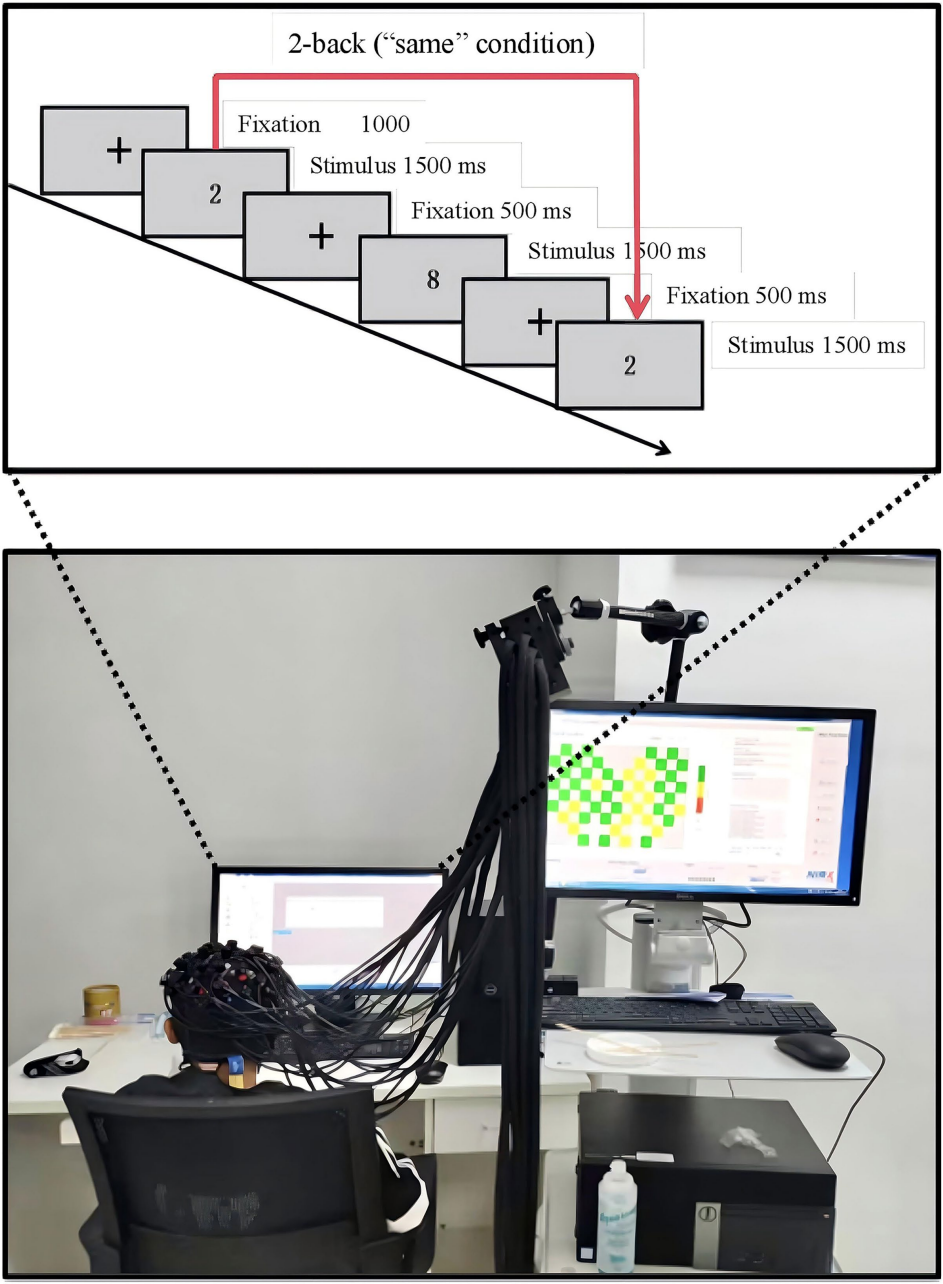
Experimental process.

Working memory was assessed using a classic 2-back task. As shown in Figure 2, a fixation cross (“+”) appeared at the center of the screen for 1,000 ms, followed by a series of numbers (1–9) presented one at a time. Each stimulus was displayed for 1,500 ms, followed by a 500 ms blank screen, resulting in a stimulus onset asynchrony of 2,000 ms. Participants were instructed to press the “F” key if the current digit matched the one presented two trials earlier, and the “J” key if it did not. No response was required for the first two digits. Reaction time and accuracy were recorded as performance measures. The practice session consisted of 10 trials. Participants were required to achieve at least 70% accuracy before proceeding to the formal test. The formal test comprised three blocks, each containing 40 trials, with a 30-s rest interval between blocks.

### 2.3 Procedure

Before the experiment began, participants were informed of the experimental purpose, procedures, schedule, and precautions, and written informed consent was obtained from all participants. All participants were required to have at least 8 h of sleep the night before the experiment. They were also asked to abstain from alcohol and stay up late for 2 days before the experiment. All testing was conducted individually in a controlled environment (sound-attenuated, temperature-regulated, and light-controlled). Mobile phones were kept turned off to avoid interference. All procedures were performed by a single experimenter. After being seated approximately 80 cm from the computer monitor, participants were fitted with the fNIRS optode cap. The experimenter calibrated the cap to ensure proper channel connectivity. Participants were then instructed to read the experimental guidelines carefully. After the practice session was completed, 180 s of resting-state cerebral blood flow data were collected before the formal test began. During the test, the experimenter continuously monitored fNIRS signal quality and recorded any anomalies (e.g., subjective details, trial, channel) for targeted processing in subsequent data analysis.

### 2.4 Data collection and analysis

#### 2.4.1 Data collection

**Figure 3:**
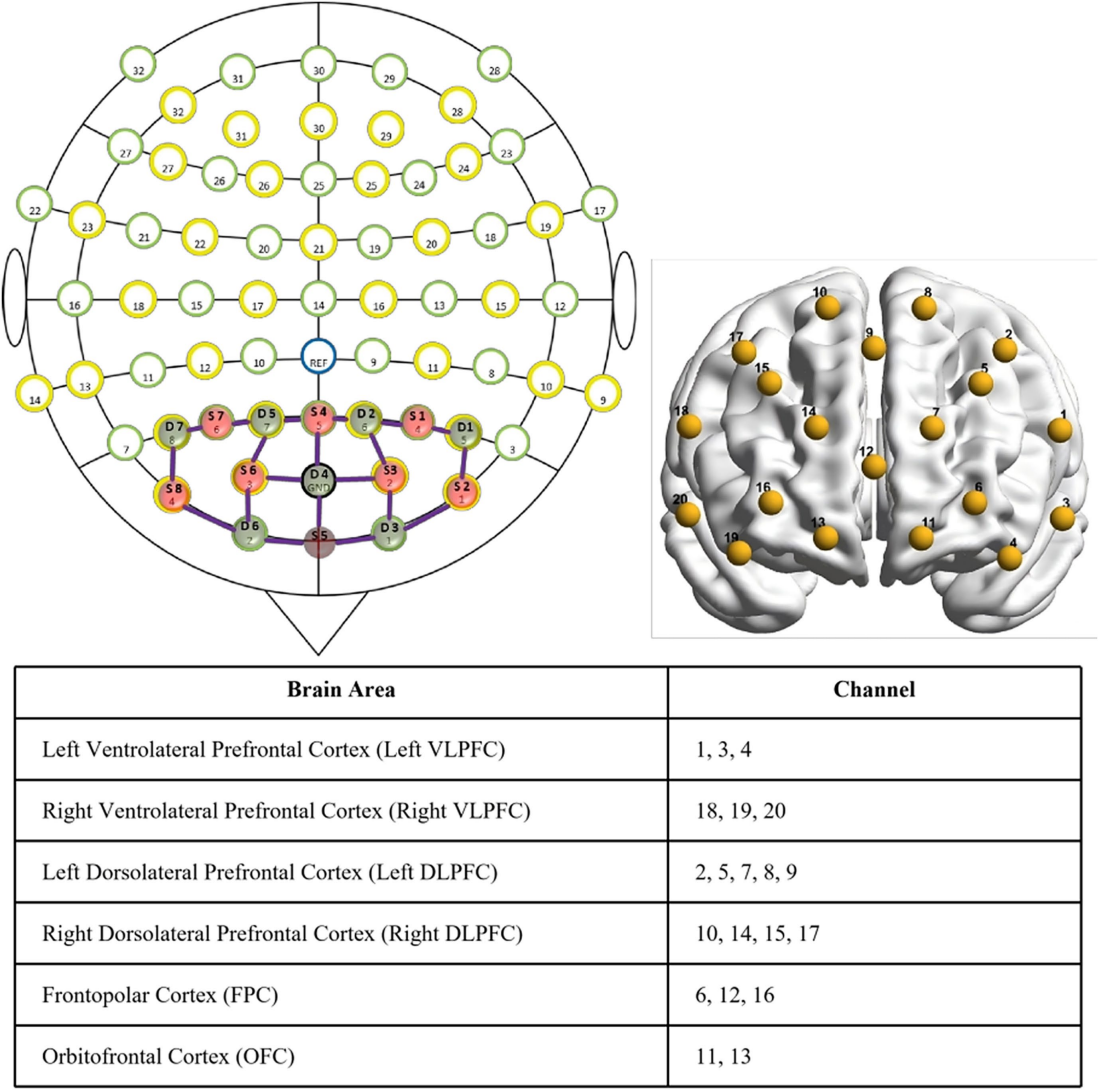
Near-infrared channel layout and corresponding Brodmann area distribution.

Behavioral data were recorded using E-Prime 3.0 software. fNIRS data were acquired using the NIRScout system (NIRx Medical Technologies, USA) to detect hemodynamic signals in the prefrontal cortex during both resting-state and task periods. Probe placement followed the international 10–20 system. The system comprised 8 emitters (wavelengths: 780 nm and 830 nm) and 8 detectors, forming 20 measurement channels over the prefrontal region. The sampling frequency was set to 10 Hz. A multichannel spatial registration method was applied to align the data to Montreal Neurological Institute (MNI) space. The near-infrared channel layout and corresponding Brodmann area distribution are illustrated in Figure 3.

#### 2.4.2 fNIRS data processing

The analysis focused on oxygenated hemoglobin (OxyHb), which provides a better signal-to-noise ratio for reflecting neural activation and is more sensitive to task responses than deoxygenated hemoglobin (Izzetoglu et al., 2007). Raw data were preprocessed using NirsLAB software (version 1.0). The preprocessing pipeline followed established best practices for fNIRS research (Yücel et al., 2021) and included:

(1) Data trimming: Removing data segments unrelated to task markers.

(2) Artifact removal: Discontinuous data caused by motion or equipment instability were removed. Spike artifacts from sudden movements or respiratory-induced drift were identified and corrected using a threshold of >0.5 mm per second in the movement sensor (when available) or a signal amplitude change >3 SD from the mean.

(3) Bandpass filtering: A bandpass filter (0.01–0.2 Hz) was applied to remove low-frequency drift and high-frequency physiological noise (e.g., cardiac and respiratory signals).

(4) Motion artifact correction: Principal component analysis (PCA) was used with two principal components retained (based on the elbow criterion in eigenvalue analysis) to reduce motion-related artifacts.

(5) Channel rejection criteria: Channels with a signal-to-noise ratio (SNR) below 5 dB or with persistent artifacts affecting more than 20% of the recording time were excluded from further analysis. In this study, no channel met this exclusion criterion.

(6) Hemoglobin calculation: OxyHb concentration changes were calculated using the modified Beer–Lambert law with a differential pathlength factor (DPF) of 6.0 for both wavelengths (780 nm and 830 nm).

(7) Baseline correction: The 2-s pre-stimulus period (from −2 to 0 s) was used as a baseline, and OxyHb changes were expressed relative to this baseline.

(8) Signal averaging: The task was divided into 3 blocks (each block = 40 trials). Because the inter-trial interval (2,000 ms) was short relative to the hemodynamic response, a block-based averaging approach was used. For each block, we calculated the mean OxyHb signal over the 2–20 s window after block onset, which corresponds to the expected peak of the hemodynamic response for sustained cognitive activation. The choice of the 2–20 s window follows previous fNIRS studies on working memory (Herold et al., 2020) and ensures that the slow hemodynamic response does not overlap across blocks (since the block duration was 80 s plus 30 s rest between blocks). The 2 s offset avoids initial vascular transients.

For each participant, OxyHb concentrations were then averaged across the three blocks, yielding mean values per channel per condition. The processed data were exported for statistical analysis.

#### 2.4.3 Data analysis

All statistical analyses were performed using SPSS 23.0. Normality was confirmed for all variables (Kolmogorov–Smirnov test, *Z* > 1.018, *p* > 0.25). To account for potential speed–accuracy trade-offs, we computed a behavioral accuracy ratio for each participant as: accuracy ratio = (mean accuracy across all trials)/(mean reaction time across all trials, in seconds), following previous studies (Chen et al., 2018). This index has the unit of per second (s^−1^) reflects processing efficiency, with higher values indicating better performance (i.e., maintaining relatively high accuracy while responding quickly). Two-way multivariate analyses of variance (ANOVAs) were conducted with exercise habits (high [HE], low [LE], no [NE]) and short video excessive usage (low [LV], moderate [MV], high [HV]) as independent variables, and working memory reaction time, accuracy, and accuracy ratio as dependent variables. To examine whether the effects of exercise habits and short video usage on prefrontal OxyHb differed across channels, we performed a three-way repeated-measures analysis of variance (ANOVA). The between-subjects factors were exercise habits (three levels: HE, LE, NE) and short video usage (three levels: LV, MV, HV). The within-subjects factor was channel (20 levels: CH1–CH20). The dependent variable was the mean OxyHb concentration change (2–20 s after block onset) for each channel. Mauchly’s test of sphericity was applied to the channel factor; if violated, degrees of freedom were corrected using the Greenhouse–Geisser method. The primary effects of interest were the main effects of each factor, their two-way interactions, and the three-way interaction (exercise habits × short video usage × channel). *Post-hoc* comparisons were conducted using Bonferroni correction when appropriate.

## 3 Results

### 3.1 Behavioral results

Reaction Time. A two-way ANOVA revealed a significant main effect of short video excessive usage on reaction time, *F*(2, 73) = 13.780, *p* < 0.001, ηp^2^ = 0.274. *Post-hoc* comparisons showed that the LV group had significantly shorter reaction times than the MV and HV groups (all *p* < 0.046), and the MV group had shorter reaction times than the HV group (*p* = 0.007). The main effect of exercise habits was not significant, *F*(2, 73) = 1.736, *p* = 0.183, ηp^2^ = 0.045, nor was the interaction, *F*(4, 73) = 0.607, *p* = 0.695, ηp^2^ = 0.032.

Accuracy. A two-way ANOVA revealed a significant main effect of exercise habits on accuracy, *F*(2, 73) = 4.900, *p* < 0.001, ηp^2^ = 0.118. *Post-hoc* comparisons showed that the HE group had significantly higher accuracy than the LE and NE groups (all *p* < 0.015), and the LE group had higher accuracy than the NE group (*p* < 0.05). The main effect of short video excessive usage was not significant, *F*(2, 73) = 0.905, *p* = 0.409, ηp^2^ = 0.024, nor was the interaction, *F*(4, 73) = 0.408, *p* = 0.751, ηp^2^ = 0.026.

**Figure 4:**
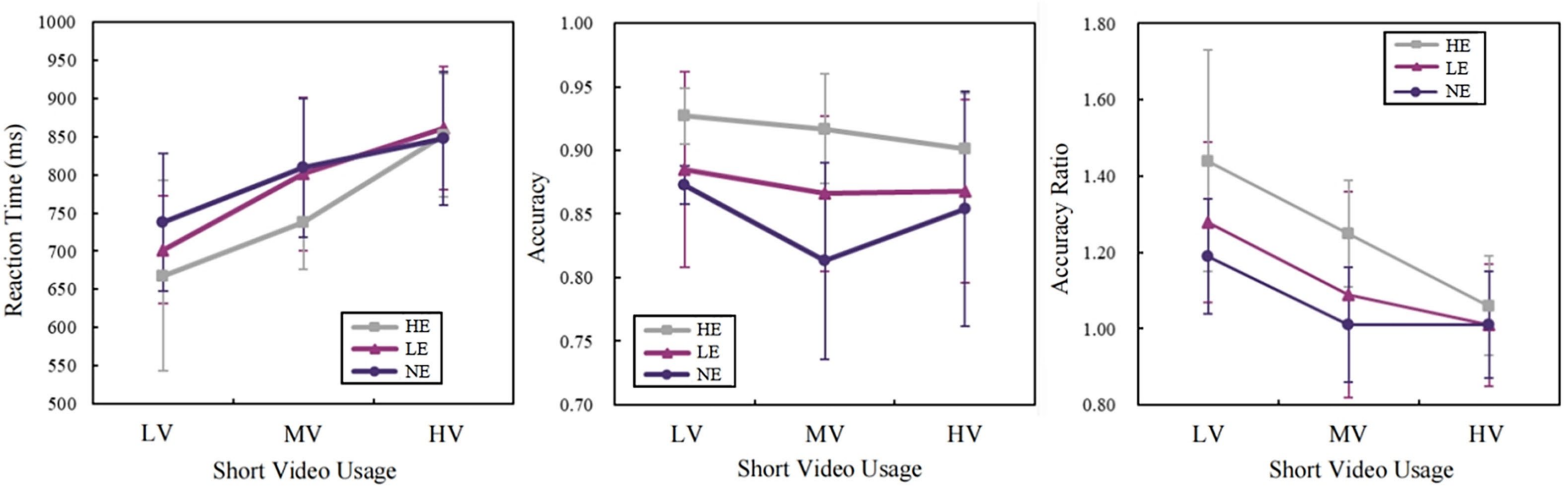
Behavioral results of the working memory task across groups. (Left) Reaction times (ms) of working memory task. (Middle) Accuracy of working memory task. (Right) Accuracy ratio of working memory task. Data are presented as individual data points (dots) with group means ± SD. HE, high exercise group; LE, low exercise group; NE, no exercise group, LV, low video group; MV, moderate video group; HV, high video group.

Accuracy Ratio. A two-way ANOVA revealed significant main effects of short video excessive usage, *F*(2, 73) = 11.029, *p* = 0.001, ηp^2^ = 0.232, and exercise habits, *F*(2, 73) = 6.143, *p* = 0.001, ηp^2^ = 0.144, on the accuracy ratio. The interaction was not significant, *F*(4, 73) = 0.866, *p* = 0.488, ηp^2^ = 0.045. Post hoc comparisons showed that for short video excessive usage, the LV group had a significantly higher accuracy ratio than the MV and HV groups (all *p* < 0.01), with no significant difference between the MV and HV groups (*p* = 0.213). For exercise habits, the HE group had a higher accuracy ratio than the LE and NE groups (all *p* < 0.035), with no significant difference between the LE and NE groups (*p* = 0.274) (Figure 4).

### 3.2 fNIRS results

The main effect of channel was significant, *F*(19, 1,387) = 1.889, *p* = 0.012, ηp^2^ = 0.025, reflecting global differences in OxyHb levels across the 20 prefrontal channels. The main effects of exercise habits and short video usage (all *p* > 0.843) and two-way interactions on OxyHb concentration were not significant (all *p* > 0.191).

**Figure 5:**
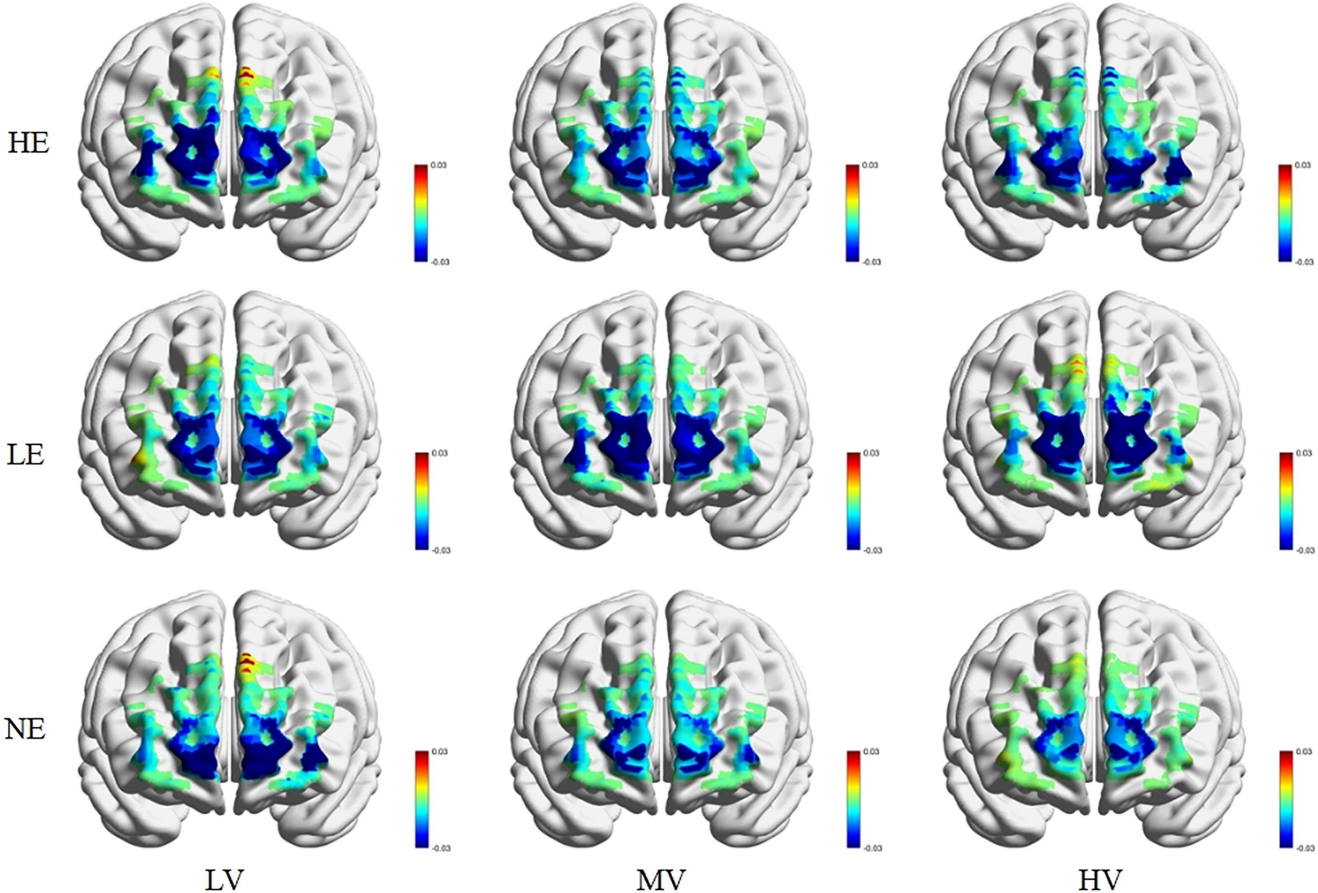
3D topographic maps of brain channel activation during the working memory task under experimental conditions. Warm colors (red/yellow) indicate stronger OxyHb activation (positive change relative to baseline); cool colors (blue/green) indicate weaker activation or deactivation (negative change relative to baseline). The color bar shows OxyHb concentration change (μM). Negative values indicate a decrease in oxygenated hemoglobin compared to the resting baseline, reflecting neural deactivation or reduced resource demand. HE, high exercise group, LE, low exercise group; NE, no exercise group, LV, low video group, MV, moderate video group, HV, high video group.

**Figure 6:**
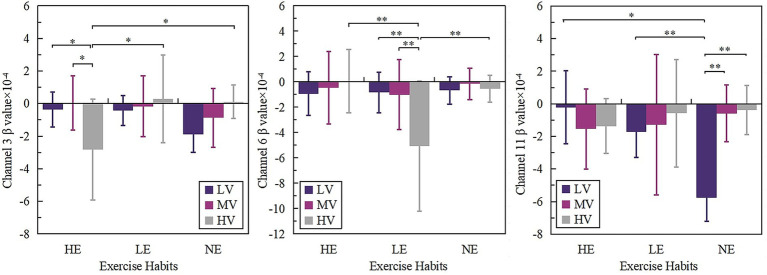
Average OxyHb changes in working memory task under experimental conditions. (Left) *β* value for channel 3 (left ventrolateral prefrontal cortex, VLPFC). (Middle) *β* value for channel 6 (frontopolar cortex, FPC). (Right) *β* value for channel 11 (orbitofrontal cortex, OFC). Data are presented as group bars with means ± SD. OxyHb values are in μM; positive values indicate activation relative to baseline, negative values indicate deactivation. HE, high exercise group; LE, low exercise group; NE, no exercise group, LV, low video group; MV, moderate video group, HV, high video group. *p* < 0.05, *p* < 0.01 for simple effects (Bonferroni-corrected).

The analysis revealed a significant three-way interaction among channel, exercise habits, and short video usage, *F*(76, 1,387) = 1.367, *p* = 0.022, ηp^2^ = 0.070. This indicates that the combined effect of exercise habits and short video usage on prefrontal OxyHb concentration changes varied significantly across different prefrontal channels. To decompose the three-way interaction, subsequent simple-effect analyses were performed and it was showed that the combination of exercise habits and short video usage differentially modulated OxyHb changes in channel 3 (left ventrolateral prefrontal cortex, VLPFC), channel 6 (frontopolar cortex, FPC), and channel 11 (orbitofrontal cortex, OFC). In channel 3, simple effects analysis revealed that within the HV group, the LE and NE groups exhibited significantly greater OxyHb in the left VLPFC than the HE group (all *p* < 0.032). Within the HE group, the LV and MV groups showed significantly greater OxyHb than the HV group (all *p* < 0.015). For channel 6, within the HV group, the HE and NE groups showed significantly greater OxyHb than the LE group in the FPC (all *p* < 0.002). Within the LE group, the LV and MV groups exhibited significantly higher OxyHb than the HV group (all *p* < 0.004). For channel 11, simple effects analysis revealed that within the LV group, the HE and LE groups exhibited significantly higher OxyHb in the OFC than the NE group (all *p* < 0.042). Within the NE group, the MV and HV groups showed significantly greater OxyHb than the LV group (all *p* < 0.006). A comparison of prefrontal cortex OxyHb during the 2-back task across experimental conditions is presented in Figures 5, 6.

## 4 Discussion

This study integrated behavioral and cerebral hemodynamic evidence to explore the brain processing mechanisms through which different levels of exercise habits relate to working memory in individuals with excessive short video use. The results revealed significant differences in the changes in behavioral performance and prefrontal activation during memory task processing among excessive short video users under different levels of exercise habits.

### 4.1 The impact of physical exercise on the behavioral performance of working memory in excessive short video users

This study focuses on the interplay between different levels of exercise habits and the degree of excessive short video use on individuals’ working memory. Findings from the analysis of the low video group revealed shorter reaction times and higher accuracy ratio than the moderate and high video groups did. Compared with the high video group, the moderate video group exhibited shorter reaction times, indicating that within the excessive short video user sample, individuals with relatively lower viewing duration had better working memory performance. A large-scale study on adolescent short video application users demonstrated that daily usage negatively predicted working memory and verbal ability (Xu et al., 2023). According to the information overload theory, when the amount of information received by an individual exceeds their information needs, processing capacity, and utilization ability, it leads to an inability to accurately select and apply effective information, resulting in cognitive overload that hinders effective information processing (Lee et al., 2016). These results align with information overload theory and suggest that excessive short video usage is linked to lower working memory performance (longer reaction times and lower accuracy ratio).

With respect to the effect of exercise habits on working memory, previous studies have reported significant differences in the extent to which different exercise habits enhance college students’ memory-related cognitive abilities (Yang et al., 2019). Using fNIRS, Zheng et al. (2022) demonstrated that acute moderate-intensity cycling significantly improves reaction time in a working memory task in young adults, accompanied by decreased prefrontal oxygenation. Additionally, research across different age groups has shown that both high and low levels of exercise habits improve working memory (Ludyga et al., 2022), with individuals engaging in higher levels of exercise demonstrating better inhibitory abilities and higher accuracy ratios in memory tasks than those with lower or no exercise habits. This finding aligns with the findings of the current study: compared with the low exercise habits (LE) group and the no exercise habits (NE) group, the high exercise habits (HE) group exhibited higher memory task accuracy and accuracy ratio, while the LE group showed higher accuracy than the NE group did. Thus, different levels of exercise habits appear to be positively related to working memory processing in individuals with excessive short video use, with higher exercise habits showing superior effects. This outcome may be partially explained by the fact that individuals with higher exercise habits engage in more varied and cognitively demanding physical activities, where physical activities in complex settings involve extensive cognitive engagement. Long-term participation in higher levels of exercise can lead to enhanced cognitive efficiency (Ludyga et al., 2018). Furthermore, while the current study primarily revealed differences in accuracy ratios, other research has shown that exercise-induced improvements in memory are more focused on reaction speed (Zimmer et al., 2017). This discrepancy may be related to response strategies in working memory experimental tasks (Postle, 2015). The LE and NE groups might have adopted strategies that sacrifice accuracy to prioritize faster reaction times. However, their reaction times, achieved at the expense of accuracy, still did not surpass those of the HE group. These findings collectively suggest that individuals with higher levels of exercise habits tend to exhibit superior working memory performance within the excessive short video user sample.

### 4.2 Physical exercise regulates the brain neural activation mechanisms of excessive short video use on working memory

Previous studies have confirmed that physical exercise could affect memory function through neuroplasticity mechanisms, inducing specific brain activation patterns (Moore and Loprinzi, 2021). The prefrontal cortex is the primary brain region involved in memory tasks (Ren et al., 2022). Physical exercise affects working memory by inducing activation changes in the bilateral frontopolar area, dorsolateral prefrontal cortex, and right premotor and supplementary cortex (Zheng et al., 2022). By analyzing the interaction effects of exercise habits and excessive short video use on prefrontal cerebral blood flow dynamics during working memory tasks, this study revealed significant OxyHb differences in CH3 (left VLPFC), CH6 (FPC), and CH11 (OFC) across groups.

Cognitive neuroscience proposes that distinct activation in specific brain regions, indicating plastic changes in brain activation patterns underlying cognitive abilities (Klingberg, 2010). The ventrolateral prefrontal cortex (VLPFC) is primarily associated with motor response selection and monitoring (Levy and Wagner, 2011), plays a decisive role in memory tasks (Evangelista et al., 2021; Tanida et al., 2012), and is closely linked to the processing of visual forms and stimulus feature information. In this study, compared with the HE group, the LE and NE groups exhibited significantly higher OxyHb levels in the left VLPFC. Combined with the behavioral results showing poorer memory task performance in the HV group, this pattern is consistent with the interpretation that this group recruited more cognitive resources during working memory processing to maintain information processing efficiency. These findings are in line with the neural compensation hypothesis of the prefrontal cortex, which posits that better cognitive performance requires the brain to activate additional pathways to compensate for cognitive decline, mobilizing other cognitive resources to offset losses in cognitive function and neural efficiency(Reuter-Lorenz and Cappell, 2008). Neural efficiency refers to the phenomenon in which the brain of brighter individuals as a whole uses fewer energy resources to cope with the task demands, by focusing the energy on smaller brain areas, those that are required to cope with the respective task demands (Neubauer and Fink, 2009). In the current study, lower prefrontal OxyHb accompanied by better working memory is interpreted as greater neural efficiency. Therefore, engaging in higher levels of exercise may effectively serve as a form of cognitive training, inducing plastic changes in brain cognition (Tang and Posner, 2009). Additionally, analysis of excessive video usage duration revealed that, within the HE group, the LV and MV subgroups exhibited significantly greater OxyHb in the left VLPFC than the HV subgroup did, together with superior reaction times and accuracy. This pattern indicates that, among individuals with high exercise habits, lower video exposure is associated with enhanced recruitment of the left VLPFC, which may contribute to better working memory performance. It suggests task-specific engagement of the VLPFC under conditions of lower cognitive load (less video overuse). Thus, among excessive short video users, different levels of exercise habits and video usage durations activate the left VLPFC in distinct ways, with higher exercise habits and lower video usage linked to more adaptive neural resource allocation (higher OxyHb level) rather than simply higher or lower efficiency.

The frontopolar cortex (FPC) is primarily involved in the decision-making process during memory tasks (Koechlin and Hyafil, 2007), with its main function being the integration of multiple goals and the formulation of optimal decision strategies (Peng et al., 2018). Analysis of the blood oxygen response in the FPC based on overuse duration revealed that, within the LE group, the LV and MV subgroups had significantly greater OxyHb in FPC than the HV subgroup, indicating that lower video exposure within low-exercisers was associated with greater cognitive resource utilization for decision-making. From the perspective of exercise habits, within the HV group, the HE participants showed significantly higher OxyHb in FPC than the LE participants. Byun et al. proposed that physical exercise can improve working memory performance, with the FPC region showing significant activation during tasks (Byun et al., 2016). Guo et al. (2016) suggested that participation in higher levels of exercise requires individuals to invest greater cognitive effort in unpredictable environments to enhance decision-making ability, and that compared with lower exercise habits, higher exercise habits demand greater neural efficiency. Because this higher activation co-occurred with better behavioral performance (HE vs. LE in the HV group), we interpret it as adaptive resource recruitment to maintain decision-making under high video-overuse conditions. Hence, higher exercise habits can activate the FPC to a greater extent when needed, supporting cognitive integration and decision-making advantages without contradicting the neural efficiency framework.

The orbitofrontal cortex (OFC) is involved primarily in the integration of complex cognitive processing during memory tasks (Burgess et al., 2007) and is associated with cortical structures that handle internal information such as equilibrium states (Miller, 2000). By comparing the OFC outcomes across exercise habit groups, it was found that the OxyHb for both HE and LE in the LV group were greater than those in the NE group. This finding aligns with previous work showing increased OFC activation after long-term yoga practice (Miyashiro et al., 2021). Importantly, when examining the NE group, we observed that the LV group showed significantly lower OxyHb in OFC than the MV and HV groups. Combined with the superior performance of the LV group, this pattern is consistent with the neural efficiency hypothesis: reduced activation accompanied by better performance reflects more economical neural processing (Neubauer and Fink, 2009). Therefore, lower OFC activation in the LV group during working memory tasks is interpreted as enhanced neural efficiency, signifying stronger cognitive integration and processing capabilities without excessive resource recruitment. This interpretation is directly aligned with the framework proposed by Neubauer and Fink (2009).

Despite the novel findings of this study, several limitations should be acknowledged. First, our sample was limited to male college students; therefore, the present findings may not generalize to females, other age groups, or clinical populations. Second, although we implemented basic experimental controls (e.g., consistent testing time, abstention from alcohol and vigorous exercise for 24–48 h), several potential confounding variables were not measured or statistically controlled. These include sleep quality, anxiety, depression, ADHD symptoms, academic stress, general smartphone and gaming use, socioeconomic background, baseline cardiorespiratory fitness, BMI, caffeine intake, and exact time of day. Future studies should include comprehensive assessments of these variables and, whenever possible, employ longitudinal or intervention designs to better isolate the effects of exercise habits and short video use. Third, we did not observe significant group differences in DLPFC activation, despite its central role in working memory. Several factors may explain this finding: (1) our study used a verbal 2-back task, and previous neuroimaging research has shown that verbal working memory primarily engages the ventrolateral prefrontal cortex (VLPFC), while spatial working memory tasks more consistently activate the DLPFC (Ren et al., 2022). (2) the cognitive load of our 2-back task may have been insufficient to elicit robust DLPFC activation in our sample of healthy young adults, who typically show ceiling effects on relatively simple working memory tasks. (3) our fNIRS probe array primarily covered the ventral and anterior prefrontal cortex, with relatively limited coverage of the most dorsal aspects of the DLPFC. (4) it is possible that the effects of exercise and short video use on DLPFC function are more subtle and would require a larger sample size or more sensitive task parameters to detect. Future studies using spatial working memory tasks and more extensive DLPFC coverage are needed to clarify this issue.Fourth, concerning the protective effects of exercise on working memory, alternative explanations should be considered. Because the study is cross-sectional, reverse causality is possible: individuals with inherently better working memory capacity may be more likely to maintain regular exercise habits and also less vulnerable to problematic short video use. Self-selection bias may also have influenced the results, as participants were not randomly assigned to exercise habit groups. Therefore, the present findings should be regarded as preliminary and hypothesis-generating rather than conclusive. Longitudinal or intervention studies are needed to establish causal directions.

## 5 Conclusion

This study examined the associations between exercise habits and excessive short video use on working memory performance and prefronta OxyHb level using behavioral measures and fNIRS in a sample of male college students. The behavioral results showed that longer excessive short video viewing duration correlates with poorer working memory in a dose-dependent manner, whereas higher levels of exercise habits are linked to superior performance. The fNIRS results revealed significant interactions in the left ventrolateral prefrontal cortex, frontopolar cortex, and orbitofrontal cortex, with individuals with higher exercise habits linked to more efficient neural activation (lower OxyHb level) and individuals with lower exercise habits requiring greater compensatory activation (higher OxyHb level). Collectively, these findings provide neurocognitive evidence that regular physical exercise are related to attenuated working memory deficits induced by excessive short video use.

## Data Availability

The original contributions presented in the study are included in the article/Supplementary material, further inquiries can be directed to the corresponding author.
